# Migraine during perimenopause

**DOI:** 10.1186/1129-2377-16-S1-A25

**Published:** 2015-09-28

**Authors:** Giovanni Battista Allais, Giulia Chiarle, Fabiola Bergandi, Chiara Benedetto

**Affiliations:** Department of Surgical Sciences, Women's Headache Center, University of Turin, Turin, Italy

Migraine affects the female sex to a greater extent than the male, with a female:male ratio of 3:1. Hormonal fluctuations during the reproductive life may influence migraine occurrence and intensity, both in a positive or negative way. Many women experience migraine approaching menopause, but the trend of migraine symptoms may vary according to the different stages of the perimenopause. If a woman is already a migraineur subject, the attacks often worsen during both the early and late phases of menopausal transition, whereas an onset of migraine is quite rare[1]. According to some authors, women with premenstrual syndrome (PMS) before menopause have an increased prevalence of migraine in late menopausal transition, and a subsequent reduction of the attacks in postmenopause[2]. The presence of PMS can be considered one of the predictors of migraine trend during the menopausal transition, since women with PMS are more sensitive to hormonal fluctuations and more prone to develop moderate to severe menopausal symptoms[3]. Hormone replacement therapy (HRT) can be used during the late premenopausal phase and the first years of postmenopause in order to counteract climacteric symptoms[4]. The effect of HRT on migraine has been investigated, either in its role of provoking or preventing the attacks. HRT should be administered continuously, without intervals, to avoid sudden estrogen deprivation and the consequent possible onset of migraine[5]. Treatment with estradiol-based gels and transdermal patches is preferable to oral formulation as it maintains constant serum hormone levels. In contrast to guidelines on the use of estroprogestinic contraceptives, migraine with aura is not an absolute contraindication to HRT when the way of administration is topical with a low dose of natural estrogens. If the aura recurs or worsens, HRT should be in any case discontinued[6]. When the effect of tibolone versus continuous combined HRT regimen in migraine is compared, a significant reduction in the hours with pain-limiting daily activities and of the amount of analgesics intake can be observed, even if there is no reduction of the days with migraine[7]. Natural menopause is associated with a lower incidence of migraine as compared with surgical menopause[8]; data on migraine prevalence in relation to the type of surgical procedure are till now unclear and contradictory[9].

## Conflict of interest

None to declare.
